# Barriers of Successful Implementation of Discharge Criteria at a Tertiary Heart Function Clinic: A Retrospective Cohort Analysis

**DOI:** 10.1016/j.cjco.2025.11.009

**Published:** 2025-11-20

**Authors:** Rami Idris, Ansh Patel, Dhruv Srikanth, Rebecca Lynn Wood, Drew McLean, Kelly McNabb, Samuel Gouett, Wendy Earle, Dianne Kirkpatrick, Sarah Culhane, Hoshiar Abdollah, Joshua Durbin, Aws Almufleh

**Affiliations:** a Faculty of Health Sciences, Queen’s University, Kingston, Ontario, Canada; b Division of Cardiology, Department of Medicine, Queen’s University, Kingston, Ontario, Canada; c Division of Cardiology, Kingston Health Sciences Centre, Kingston, Ontario, Canada; d Faculty of Nursing, University of Toronto, Toronto, Ontario, Canada

**Keywords:** heart failure, discharge protocols, outpatient care

## Abstract

**Background:**

Most heart function clinics cannot absorb their high volume of referrals. The effectiveness of clinic discharge protocols to offload stable patients is understudied. We examined predictors and barriers of implementing discharge criteria at our tertiary heart function clinic.

**Methods:**

This is a retrospective analysis of discharge protocol implementation between August 1, 2023 and March 31, 2024. Outcomes were discharge and rates of acute care utilization within 6-months postdischarge.

**Results:**

Of 153 patients reviewed, 92 were suitable for discharge, but only 56 of 92 (60.9%) were discharged. Discharge failure was associated with the following: atrial fibrillation (66.7% not discharged vs 30.4% discharged; *P* < 0.001); ejection fraction < 50% at the last visit (77.8% not discharged vs 51.8% discharged; *P* = 0.012); worse kidney function (initial visit creatinine 101.0 vs 86.5 μmol/L for those discharged not discharged, respectively; and at last visit, 106.5 vs 99.0 μmol/L); and provider experience < 10 years (for 36.1% not discharged vs 16.1% discharged; *P* = 0.028). Reasons cited for discharge failure were that providers were awaiting an extra echocardiogram (48.6%) or coordinating with other cardiac care teams (27.0%). In the 6 months following discharge, 3 patients (5.4%) visited the emergency department for heart failure, 1 patient (1.8%) was hospitalized for heart failure, and 1 patient (1.8%) passed away from cancer. Three of 5 adverse outcomes were judged to be unavoidable even if clinic follow-up had been continued.

**Conclusions:**

Discharge rate from our clinic is suboptimal, which impedes timely care for new referrals. We identified several patient- and provider-specific barriers to discharge. More studies are needed to explore this important area.

## Résumé

**Contexte:**

La plupart des cliniques spécialisées dans la fonction cardiaque sont incapables d’absorber le volume important de patients qui leur sont adressés. L’efficacité des protocoles de sortie de l’hôpital visant à donner leur congé aux patients dont l’état est stable pour désengorger les cliniques n’a pas été suffisamment étudiée. Nous avons examiné les facteurs de prédiction et les obstacles à l’application des critères de sortie de l’hôpital dans notre clinique de soins tertiaires spécialisée dans la fonction cardiaque.

**Méthodologie:**

Il s’agit d’une analyse rétrospective évaluant la mise en œuvre du protocole de sortie de l’hôpital entre le 1^er^ août 2023 et le 31 mars 2024. Les paramètres évalués étaient le congé de l’hôpital et le taux d’utilisation des soins actifs dans les 6 mois suivant le congé de l’hôpital.

**Résultats:**

Parmi les 153 patients examinés, 92 étaient aptes à recevoir leur congé de l’hôpital, mais seuls 56 d’entre eux (60,9 %) l’ont reçu. L’échec du congé de l’hôpital a été associé à la fibrillation auriculaire (66,7 % des patients demeurés à l’hôpital vs 30,4 % des patients ayant quitté l’hôpital; *p* < 0,001), à une fraction d’éjection < 50 % à la dernière visite (77,8 % des patients demeurés à l’hôpital vs 51,8 % des patients ayant quitté l’hôpital; *p* = 0,012), à une détérioration de la fonction rénale (taux de créatinine à la visite initiale de 101,0 μmol/L chez les patients ayant quitté l’hôpital vs 86,5 chez ceux demeurés à l’hôpital, et 106,5 vs 99,0 μmol/L lors de la dernière visite) et à l’expérience du professionnel de la santé < 10 ans (36,1 % des patients demeurés à l’hôpital vs 16,1 % des patients ayant quitté l’hôpital; *p* = 0,028). Les motifs évoqués pour expliquer l’échec du congé de l’hôpital sont les suivants : le professionnel de la santé attendait une échocardiographie de confirmation (48,6 %) ou la coordination avec d’autres équipes de soins cardiaques (27,0 %). Dans les 6 mois suivant le congé de l’hôpital, 3 patients (5,4 %) se sont rendus à l’urgence pour une insuffisance cardiaque (IC), 1 patient (1,8 %) a été hospitalisé pour une IC et 1 patient (1,8 %) est décédé d’un cancer. Trois événements indésirables sur cinq ont été jugés inévitables, même si le suivi clinique s’est poursuivi.

**Conclusions:**

Le taux de congé de l’hôpital est sous-optimal dans notre clinique, ce qui nous empêche d’offrir des soins en temps opportun aux autres patients qui nous sont adressés. Nous avons ciblé plusieurs obstacles au congé de l’hôpital, propres aux patients et aux fournisseurs de soins. D’autres études s’imposent pour explorer ce domaine important.

Nearly 828,000 Canadians live with heart failure (HF), a chronic disease with a heavy symptom burden, significant impact on quality of life, and an estimated 50% mortality within 5 years of diagnosis.^1^^,^^2^ Heart function clinics (HFCs) are subspecialty centres that offer timely care to optimize and stabilize patients with HF.^3^ They have been shown to be cost-effective and to lead to reduction in all-cause mortality and hospitalizations for HF. However, the number of specialized HFCs is not sufficient to meet the needs of the those with HF given its high prevalence in the population.^4^ In Ontario, one estimate is that HFCs do not have the capacity for more than 10% of the HF patients in the province.^4^

Currently, most HFCs struggle with long waitlists and the inability to absorb the high volume of referrals they receive.^5^ The Canadian Cardiovascular Society (CCS) 2017 guidelines published broad criteria for “safe” discharge, but prior survey studies reported that > 30% of HFCs admit to having poor adherence to the discharge criteria.^5^^,^^6^ This finding is unfortunate, as effective discharge of clinic patients can lead to substantial improvement in clinic capacity and reduction in wait times. Of particular importance, the patient- and provider-level barriers to discharging eligible patients from HFCs remains poorly understood. At our clinic, we developed a discharge protocol based on guideline recommendations and clinical experience. This study aims to define rates of discharge and identify barriers to discharge of stable patients from the clinic. Results will be crucial to guide improvements in the clinic discharge process, increase the rate of discharge, and improve clinic capacity.

## Methods

### Setting and patient population

The HFC at Kingston Health Sciences Centre (KHSC) serves a catchment area of 500,000 people and conducts > 2000 visits per year. The practice model is primarily nurse practitioner-driven, with one HF physician attending each in-person clinic to independently see new patients and review other cases, with the 5 nurse practitioners running the clinic.

**Figure 1:**
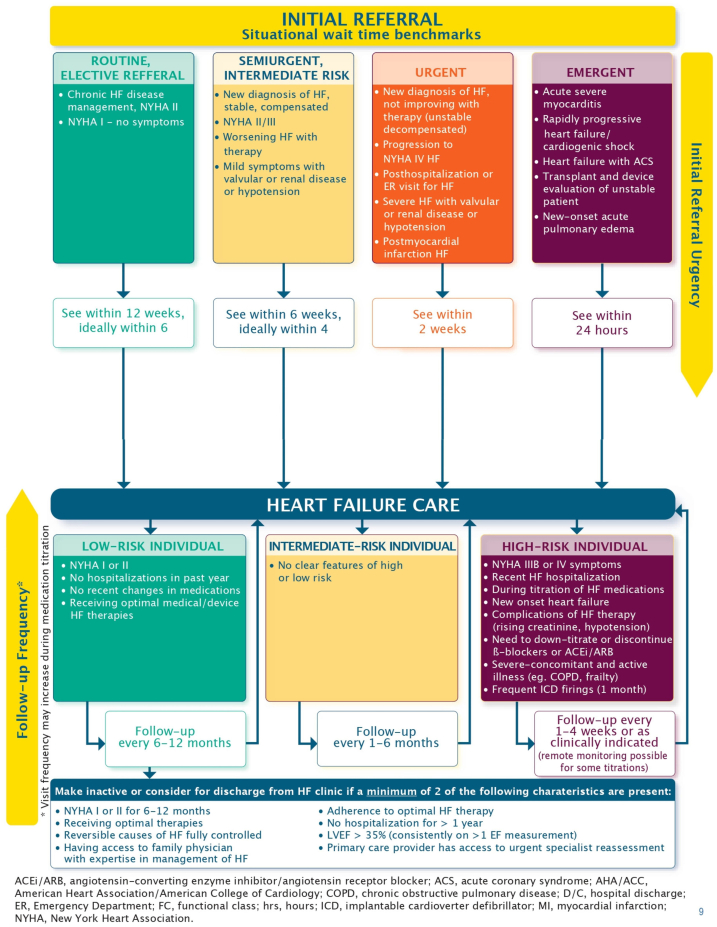
Canadian Cardiovascular Society treatment algorithm and discharge criteria for heart failure (HF). ACEi, angiotensin-converting enzyme inhibitor; ACS, acute coronary syndrome; EF, ejection fraction; ER, emergency room; ARB, angiotensin receptor blocker; COPD, chronic obstructive pulmonary disease; ICD, implantable cardioverter defibrillator; LVEF, left ventricular ejection fraction; NYHA, New York Heart Association.

**Figure 2:**
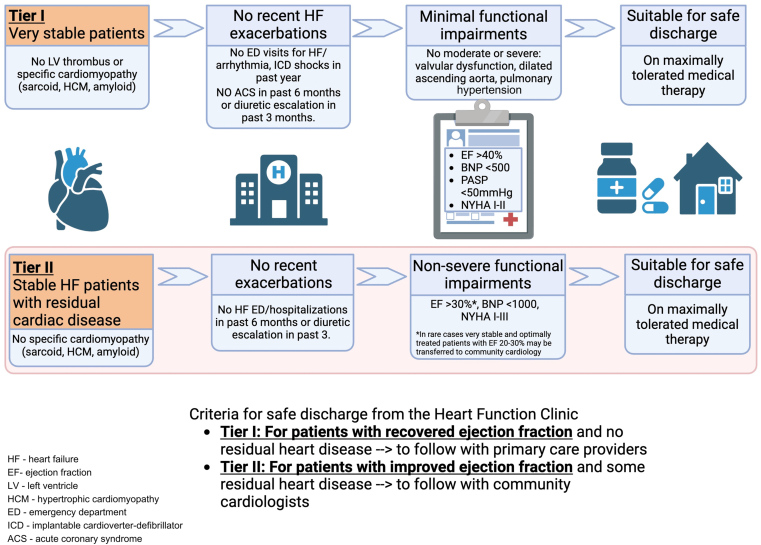
Kingston Health Sciences Centre Heart Function Clinic discharge algorithm. ACS, acute coronary syndrome; BNP, brain natriuretic peptide; ED, emergency department; EF, ejection fraction; HCM, hypertrophic cardiomyopathy; HF, heart failure; ICD, implantable cardioverter defibrillator; LV, left ventricle; NYHA, New York Heart Association; PASP, pulmonary artery systolic pressure.

In response to the accumulating referrals and increasing wait times, we customized the CCS discharge criteria to our local setting and developed a 2-tier discharge system. This system was developed during multiple meetings of the HFC team members. CCS guidelines (Fig. 1) were used as a baseline, followed by input from clinic staff with varying levels of experience. Specific cardiomyopathies (for example, amyloidosis and sarcoidosis) were considered exclusions for discharge, as they pose an increased risk of HF exacerbation and require close and ongoing monitoring. Similarly, given that B-type natriuretic peptide (BNP) levels have been shown to be a reliable predictor of patient outcomes, they were embedded in the discharge criteria.^6^^,^^7^ Naturally, practitioners are expected to make decisions based on a combination of the criteria and their own clinical judgement. For example, although it is not explicitly noted in the discharge criteria, providers were aware that any patients deemed unstable or without avenues for ongoing care (patients without a primary care provider [PCP]), even if they were deemed “suitable for discharge according to the clinic criteria,” should be retained in the clinic until adequate follow-up support is secured. The discharge criteria categorized patients according to their readiness for discharge and their degree of need for ongoing care. Tier I is a track for very stable patients who can be discharged safely to the care of their PCP. Tier II is for patients who no longer need HFC services but require cardiologist follow-up to manage residual cardiac disease (eg, moderate valvular disease or aortic root dilatation that require annual monitoring); these patients are sent back to their primary cardiologist, if available, and if not, they are referred to a new cardiologist for continued care (Fig. 2). Our discharge criteria were discussed with all HF clinicians at a group meeting and then were circulated via e-mail. At the time of implementation, incorporating any criteria in the electronic medical records was not feasible.

This retrospective cohort analysis was a quality improvement initiative to determine the rate of effectively implementing the discharge criteria and barriers to their implementations. Ethical approval was obtained from Queen’s University Health Sciences Research Ethics Board. This analysis is retrospective and reports the impact of this initiative; thus, consent from patients was not required.

### Data collection

Two independent reviewers retrospectively examined the charts of all patients who had their 3rd visit at the KHSC HFC between Aug 1, 2023 and March 31, 2024. To capture cases for which deliberate use of the discharge criteria can be most impactful, analysis was restricted to patients with ≥ 3 visits at the KHSC HFC. This population was sufficiently complex to necessitate the use of the discharge criteria. Patients discharged after their first or second visit typically are very stable or lack an HF diagnosis; they therefore do not require use of the discharge criteria. Reviewers extracted demographic, clinical, laboratory, and imaging characteristics at the initial and most recent clinic visits (Table 1). The reviewers also examined clinic notes at any follow-up visits to date, to collect potential reasons for failure to discharge if they were given in the provider notes. Provider characteristics, including their role (physician vs nurse practitioner) and experience level (measured by number of years in practice) also were documented.

**Table 1:** Demographic statistics

Characteristic	Discharged n = 56	Not discharged n = 36	All patients N = 92	*P*
Female sex	21 (37.5)	11 (30.6)	32 (34.8)	0.495
Hypertension	36 (64.3)	22 (61.1)	58 (63)	0.758
Diabetes mellitus	10 (27.8)	17 (30.4)	27 (29.3)	0.791
Chronic kidney disease	4 (7.1)	2 (5.6)	6 (6.5)	1.000
Myocardial infarction	22 (39.2)	10 (27.8)	32 (34.8)	0.258
Coronary artery disease	30 (53.6)	18 (50.0)	48 (52.2)	0.738
Atrial fibrillation	17 (30.4)	24 (66.7)	41 (44.6)	**0.001**
Ventricular tachycardia	4 (11.1)	4 (7.1)	8 (8.7)	0.707
Valvular heart disease∗	34 (60.7)	21 (58.3)	55 (59.8)	0.820
Stroke	9 (16.1)	1 (2.8)	10 (10.9)	0.082
Transient ischemic attack	4 (7.1)	0 (0.0)	4 (4.3)	0.153
Has a primary care provider	49 (87.5)	30 (83.3)	79 (85.9)	0.576
Has a cardiologist	4 (7.1)	2 (5.6)	6 (6.5)	1.00
Any ED visits within the past 12 months of the 1st visit	36 (64.3)	27 (75.0)	63 (68.5)	0.280
Any HF hospitalizations within the past 12 months of the 1st visit	33 (58.9)	21 (58.3)	54 (58.7)	0.955

Values are n (%), unless otherwise indicated. N = 92. Bodface indicates significance.

ED, emergency department; HF, heart failure.

∗ Defined as ≥ mild-to-moderate disease in any heart valve.

By applying the clinic’s established discharge algorithm (Fig. 2), patients were categorized as being ineligible for discharge or eligible for tier I or tier II discharge. The primary outcome was rate of discharge (defined as whether those patients deemed to be suitable for discharge were in fact discharged by the clinic providers). Discharge was defined as explicit documentation of care providers stating that the patient was being discharged from the clinic, and a lack of subsequent follow-up visits to the HFC within the following 6 months. The secondary outcome was overall safety of the discharge algorithm (defined as a composite of emergency department (ED) visits, hospitalizations, or mortality within 6 months of discharge). For discharged patients, reviewers documented the occurrence and frequency of these events, and they reviewed event documentation to determine if the reasons for occurrence of these events were HF-related, and whether they could have been avoided through continued monitoring in the clinic.

### Statistical analysis

Descriptive summary statistics were reported, as appropriate, and a comparative analysis was performed to identify factors significantly associated with discharge. Categorical variables were compared via χ^2^ test , and numeric variables were compared via the independent *t*-test and the Mann-Whitney *U* test. SPSS Statistics, version 29 (IBM, Armonk, NY) was used for data analysis. To account for missing data, tests were run with pairwise exclusion.

## Results

Of the 153 HF patients who had at least 3 visits at our tertiary HFC in Kingston, Ontario, during the study period, 92 (60.1%) were deemed suitable for discharge, but only 56 of 92 patients (60.8%) were actually discharged (the discharge rate was 75.0% among tier I and 53.3% among tier II suitable patients, *P* = 0.043). Demographic (Table 1) and clinical (Table 2) characteristics of patients suitable for discharge, comparing those who were discharged vs those who were not.

**Table 2:** Clinical, laboratory, and imaging statistics

	N	Discharged	Not discharged	All patients	*P*
Provider experience ≥10 y	92	47 (83.9)	23 (63.9)	70 (76.1)	**0.028**
Patient seen by nurse practitioner (as opposed to physician)	92	55 (98.2)	34 (94.4)	89 (96.7)	0.559
Number of phone visits	92	7.0 (4.0–10.0)	12.0 (7.0–18.0)	8.0 (5.0–13.0)	**0.001**
EF at initial clinic visit, %	92	31.5 (23.0–39.0)	31.5 (27.0–38.0)	31.5 (24.3–38.0)	0.785
EF at most recent clinic visit, %	92	48.2 ( ± 9.7)	43.8 ( ± 8.8)	46.5 ( ± 9.6)	**0.032**
BNP pg/mL at initial visit, pg/mL	85	238.5 (83.0–907.0)	203.0 (74.0–401.0)	232.0 (75.0–601.5)	0.490
BNP at most recent visit, pg/mL	84	122.5 (52.0–276.5)	156.5 (91.5–312.5)	144.0 (64.5–287.0)	0.138
Creatinine at initial visit, μmol/L	92	86.5 (72.0–112.5)	101.0 (87.5–116.5)	92.5 (75.0–114.8)	**0.036**
Creatinine at final visit, μmol/L	91	99.0 (70.0–116.5)	106.5 (91.5–131.0)	102.0 (81.0–124.0)	**0.048**
Months from first to last visit	92	18.00 (11.00–28.50)	30.00 (23.00–49.50)	23.00 (13.00–33.50)	< 0.001

Values are n (%), median (interquartile range), or mean ± standard deviation, unless otherwise indicated. Boldface indicates significance.

EF, ejection fraction; BNP, B-type natriuretic peptide.

### Role of comorbidities, ejection fraction, and kidney function

Patients with atrial fibrillation (AF) were less likely to be discharged; 66.7% of those not discharged had AF, compared to 30.4% among those who were discharged (*P* < 0.001). Other clinical factors, including hypertension, type II diabetes, smoking history, stroke, transient ischemic attack, and previous coronary artery disease, were not associated with a statistically significant difference in discharge outcomes (*P* > 0.05).

Regarding laboratory and imaging measures, patients who were discharged had a higher ejection fraction (EF) (mean EF, 48.2% vs 43.8%, *P* = 0.032) at the discharge visit. Discharge also was associated with lower levels of creatinine at both the first clinic visit (mean 86.5 vs 101.0 μmol/L; *P* = 0.036) and the last clinic visit (mean, 99.0 vs 106.5 μmol/L; *P* = 0.048), comparing discharged patients with those who were not discharged, respectively.

### Role of guideline-directed medical therapy

Notably, sodium glucose cotransporter-2 inhibitor (SGLT2i) use at the first visit was associated with higher discharge rates (24 patients (42.9%) of those discharged vs 6 patients (16.7%) of those not discharged, *P* = 0.009). No significant differences occurred in the use of other guideline-directed medical therapy (angiotensin-converting enzyme inhibitors [ACEis], angiotensin II receptor blockers [ARBs], angiotensin receptor neprilysin inhibitors [ARNIs], beta-blockers, and mineralocorticoid receptor antagonists [MRAs]) between those who were discharged vs those who were not discharged (*P* > 0.05).

### Impact of follow-up duration and whether patients have a PCP or a cardiologist

Discharged patients had a significantly lower median number of heart function telephone visits compared to the number for patients not discharged (7.0 [interquartile range = 4.0-10.0] vs 12.0 (interquartile range = 7.0-18.0); *P* < 0.001). They also had shorter durations of follow-up care than patients not discharged (median, 18 vs 30 months; *P* < 0.001).

Access to a PCP did not affect discharge outcomes, with 87.5% of discharged patients having a PCP, compared to 83.3% of those not discharged (*P* = 0.576). Only a few patients in this study had a cardiologist (6 of 92)—which did not significantly impact discharge rates—7.1% of patients discharged vs 5.6% of patients not discharged; *P* = 1.00.

### Impact of clinic providers’ roles and years of experience

As for HF care provider characteristics, provider role (nurse practitioner or medical doctor) had no statistically significant impact on discharge rates. However, early-to-mid career status (defined as < 10 years in practice) was associated with lower rates of discharge—40.9% in those treated by early-to-mid-career providers vs 67.1% in those treated by more experienced providers; *P* = 0.028.

To investigate reasons for failure to discharge, a thematic analysis of clinic notes was conducted (Table 3). This analysis revealed that the desire to repeat echocardiograms (1 more time) to confirm the improvement of left ventricular function (in 18 of 37; 48.6%), and coordinating care with other cardiac care teams (arrhythmia, surgery, structural heart team; 17 of 37; 45.9%) were the most-cited barriers for discharge of patients who otherwise met discharge criteria.

**Table 3:** Thematic analysis of reasoning given for not discharging patients

Thematic analysis category	N = 37
1**)** Awaiting repeat echocardiogram despite most recent echocardiogram showing left ventricular ejection fraction within limit suitable for discharge	18 (48.6)
2**)** Awaiting electrophysiology appointment and/or consultation for arrhythmia or implantable device	10 (27.0)
3**)** Awaiting additional cardiac evaluations unrelated to heart failure diagnosis and/or management	7 (18.9)
4**)** Not discharged, due to lack of primary care provider	1 (2.7)
5**)** Not discharged, due to noncardiac reason (eg, infection, malignancy, etc.)	1 (2.7)

Values are n (%), unless otherwise indicated.

### Safety outcomes

In the 6 months after discharge, 5 patients (9%) developed a safety outcome. Three (5.4%) patients visited the ED for HF; none visited the ED more than once; 1 patient (1.8%) was hospitalized for HF; and 1 patient (1.8%) passed away from cancer. Closer investigation showed that 3 of 5 adverse outcomes in these patients occurred secondary to a cause that could not be avoided by further follow-up in the HF clinic (ie, 2 HF exacerbations deemed secondary to sepsis and acute myocarditis, and 1 death due to cancer). Only 2 patient outcomes potentially could have been avoided; 1 of these was a patient who had low adherence to a low-sodium diet, which could have been avoided through phone calls to educate and reinforce lifestyle modifications. All discharged patients were appropriately referred to a community cardiologist after discharge.

## Discussion

This study is the first to investigate barriers to implementing discharge protocols at a multidisciplinary HF clinic. We herein report that only 60.9% of patients who were deemed ready for discharge by our criteria were actually discharged. Understanding and addressing barriers to successful discharge will have a substantial impact on HFC capacity, as more successful discharges result in more space to accommodate new patients and decrease wait times.

### Factors associated with failure to discharge

#### Clinical complexity

Clinical complexity played a role in discharge decisions, as patients with residual cardiac disease (deemed suitable for tier II discharge) were significantly less likely to be discharged than the stable patients deemed suitable for tier I discharge. Other surrogates for clinical complexity supported this finding, such as the lower EF, poorer kidney function, higher frequency of telephone visits, and longer duration of follow-up that was observed in the group that was not discharged.

Comorbid AF emerged as a significant factor impacting failure to discharge, likely because it compelled HF clinicians to hold on to these patients, considering the high symptomatic burden of AF and the need for specialized tests (eg, Holter monitors).^8^ Previous work corroborates our findings; in a study aimed to identify barriers to discharge from subspecialty internal medicine clinics, providers cited the presence of comorbidities that may result in frequent exacerbations and hospitalizations as one of the key reasons that dissuades physicians from discharging patients.^9^

#### Healthcare provider education

Thematic analysis indicates that the most common reason associated with failure to discharge was providers’ wishes to repeat another echocardiogram to confirm stability, even though those patients had already met the discharge criteria. This finding indicates that some care providers may not be comfortable with the current discharge protocol. Further, patients who were seen by a clinic provider with ≥ 10 years of experience were significantly more likely to be discharged (67.1%) than were those seen by less-experienced providers (40.9%; *P* = 0.028). More-experienced providers may be able to balance the drive to keep patients longer out of extra caution with the wider responsibility to allocate resources fairly to the unseen patients on the waitlist.^9^ The demonstrated safety of this discharge protocol hopefully will serve to reassure providers with less clinical experience of the safety of the discharge criteria and the need to implement them.

Patients with a longer follow-up duration were less likely to be discharged. Although follow-up duration may be a surrogate for clinical complexity, another possibility is that patients with longer follow-up duration develop roots of trust and comfort with the clinic staff, which could impede their discharge. As is the case with any long-term relationship, termination can be difficult—patients often insist on remaining in follow-up care, and providers feel very responsible for not “abandoning” these patients. This sentiment was noted in a previous study investigating barriers to discharge in an ambulatory internal medicine clinic; providers cited patients’ anxiety regarding postdischarge follow-up care to be a common barrier.^9^

A common fallacy is the sense that keeping the patient enrolled in clinic with very infrequent follow-up (once per 12 or 18 months) is essentially the same as full discharge from the clinic. This belief is untrue for 2 reasons. First, in a clinic with > 600 active patients and the capacity to conduct 2000 visits per year, a once-per-year appointment for every patient will instantly block 30% of the clinic capacity that year. Second, when stable patients remain attached to the clinic, they invariably use its resources but not always for HF-specific reasons.

### Safety of discharge

Review of patients’ outcomes after discharge from the clinic revealed only a minimal number of HF-related events. Although the risk of acute care needs can never be completely eliminated (even by rigorous clinic follow-up care), our protocol seems to strike a good balance between effective discharge and safe outcomes among those appropriately discharged.

### Implications and lessons learned

This study is the first to report on barriers to implementing a discharge protocol at an HFC. Guided by this study, we developed the following strategies to improve discharge success at our clinic and provide guidance for other centres that wish to implement discharge criteria:

1. We developed a network with general cardiology groups to facilitate the handoff of tier II patients once they “graduate” from needing HFC services. This approach serves to reassure patients and their PCPs that their cardiac needs will be met.
2. We will transfer HF patients whose only cardiac needs are for AF to the local arrhythmia clinic, which is best equipped to manage these patients and organize specialized interventions for AF. In parallel, this collaboration facilitates the transfer of comorbid AF and HF patients requiring HF-specific care to the HFC, while ensuring that patients’ care needs are delegated to the most suitable provider.
3. To prepare patients for potential discharge, our providers prepared the following sample statement: “You will be getting an ultrasound of the heart next month. If the heart pumping percentage improves, you will have graduated from our clinic. I will call to inform you of the good news and that you no longer need HF clinic follow-up. Your primary care provider will receive a letter explaining when to further repeat the heart tests and reasons to adjust the medicines or send you back for our assessment.”
4. The safety results of this study will be communicated to clinic staff to foster more trust in the discharge protocol. We also will discuss the barriers that must be overcome to further improve discharge rates.
5. Future iterations of our discharge criteria will explicitly incorporate instructions regarding patients who are suitable for discharge but do not have a clear avenue for follow-up care.

Future research should explore the role of technology and artificial intelligence, to use data from electronic medical records to flag patients suitable for discharge, and then remind the providers of this status when they access their charts. Insights into the safety outcomes of patients who were not discharged is also an important area for future studies,

### Limitations

Although our study adds to the literature in an understudied field, we acknowledge several limitations. First, this study was conducted at a single centre and was relatively small, which may have impacted the statistical power in our analyses. Second, the follow-up period after discharge from the clinic was only 6 months, and is therefore not representative of long-term patient outcomes. Future research examining this topic should use a longer follow-up window to ascertain the long-term safety of discharge from an HFC. Further, because follow-up outcome ascertainment relied on retrospective review of electronic medical records within our institution, some safety outcomes may have occurred outside our catchment area and therefore were not captured. Additionally, for our discharge criteria to become widely adaptable, we should incorporate the requirement that appropriate follow-up be arranged before the patient is discharged, particularly in cases in which the patient does not have a PCP. In addition, although we used BNP in our discharge algorithm, given its widespread use at our institution, we recognize that N-terminal pro-B-type natriuretic peptide (NT-proBNP) is more commonly utilized in Canada and thus future studies should incorporate it into their algorithms. Finally, this study did not include patient perspectives on discharge from the clinic, which would have provided valuable insights.

## Conclusion

Consistent with the literature, the rate of discharge from our HFC was only 60.9%, which is suboptimal. Addressing comorbidities and care-practitioner education, and creating avenues to better accommodate clinical complexity, emerged as key areas in which to make adjustments to increase the rate of discharge of stable HF patients. In turn, such changes will increase the capacity of HFCs to see new referrals and allow more patients to benefit from their services.

## References

[1] Government of Canada Canadian Chronic Disease Surveillance System (CCDSS). https://www.canada.ca/en/public-health/services/publications/diseases-conditions/heart-disease-canada.html.

[2] Lawrence S. Heart failure in Canada: complex, incurable and on the rise. https://www.heartandstroke.ca/what-we-do/media-centre/news-releases/heart-failure-in-canada-complex-incurable-and-on-the-rise.

[3] Tran K., Butcher R. (2021). CADTH Health Technology Review.

[4] Kugathasan L., Francis T., Rac V.E. (2021). A 2020 environmental scan of heart failure clinics in Ontario. CJC Open.

[5] Virani S.A., Zieroth S., Bray S. (2020). The status of specialized ambulatory heart failure care in Canada: a joint Canadian Heart Failure Society and Canadian Cardiovascular Society heart failure guidelines survey. CJC Open.

[6] Ezekowitz J.A., O'Meara E., McDonald M.A. (2017). 2017 comprehensive update of the Canadian Cardiovascular Society Guidelines for the Management of Heart Failure. Can J Cardiol.

[7] Kociol R.D., Horton J.R., Fonarow G.C. (2011). Admission, discharge, or change in B-type natriuretic peptide and long-term outcomes: data from Organized Program to Initiate Lifesaving Treatment in Hospitalized Patients with Heart Failure (OPTIMIZE-HF) linked to Medicare claims. Circ Heart Fail.

[8] Rosner G.F., Reiffel J.A., Hickey K. (2012). The concept of "burden" in atrial fibrillation. J Atr Fibrillation.

[9] Halani S., Melvin L., Cavalcanti R.B. (2024). Determining readiness: a qualitative study and educational framework on discharge practices in ambulatory internal medicine. Can J Gen Intern Med.

